# An Observational Study of Physicians' Workflow Interruptions in Outpatient Departments in China

**DOI:** 10.3389/fpubh.2022.884764

**Published:** 2022-04-29

**Authors:** Ximin Zhu, Yinhuan Hu, Liuming Wang, Dehe Li, Xiaoyue Wu, Shixiao Xia, Siyu Cheng

**Affiliations:** ^1^School of Medicine and Health Management, Tongji Medical College, Huazhong University of Science and Technology, Wuhan, China; ^2^Tongji Hospital, Tongji Medical College, Huazhong University of Science and Technology, Wuhan, China

**Keywords:** outpatient, workflow interruptions, interdepartmental difference, observational study, occupational environment safety, human factor

## Abstract

**Background:**

Workflow interruptions are frequent in hospital outpatient clinics. Eventually, not only reducing the work efficiency and quality, but also further threatening patient safety. Over the last 10–15 years, research on workflow interruptions in inpatient care has increased, but there is a lack of research on the interruptions in outpatient clinics. The present study aimed to study the differences in physicians' workflow interruptions among outpatient departments in the tertiary hospital in China.

**Methods:**

In a tertiary hospital, a standardized observational study of 32 doctors' workflow in outpatient department of four typical clinical specialties was conducted. The record of workflow interruptions was based on a self-made observation instrument after verifying its reliability and validity. Linear regression methods were used to assess outpatient characteristics as predictors of the number of interruptions. The Kruskal-Wallis test was used to analyze the difference about the duration of interruptions among specialties, and the Chi-Square Test was used to examine the sources of interruptions among different specialties, to determine whether interruption source is associated with specialty.

**Results:**

The number of patients was the significant independent predictor of the number of interruptions (*p* < 0.001). In terms of work tasks being interrupted, the highest interruption rate occurred when physicians were asking health history: 19.95 interruptions per hour. The distribution of interruption sources among the four clinical specialties were statistically different (*X*^2^ = 16.988, *p* = 0.049).

**Conclusion:**

The findings indicate that physicians' workflow interruptions are connected with many contents in the work system. Further emphasis should be placed on the effective application of hospital management measures in an interrupted environment to promote a safe and efficiency outpatient care.

## Introduction

It is common that physicians are interrupted frequently in the hospitals. Workflow interruptions divert physician's attention to the interrupting event and away from the current task (1), which may in turn affect the quality and efficiency of the services, and consequently pose a risk to the patient safety (2, 3). Interruption is defined as an event that diverts the attention of a doctor from a task at hand; when people resume work after an interruption, they often find themselves distracted and unable to concentrate (4). Many studies have shown that interruptions occur frequently in different medical settings (5–7), but studies on interruption in outpatient settings are relatively lacking.

The outpatient setting is dynamic. Outpatient physicians often need to fully consider the patient's condition. If there is an unreasonable interruption in the work process, it will interrupt the doctor's thinking of diagnosis and treatment and affect the quality of medical care. Interruption of physician processes is not always negative (8), but positive (9–11). One should also look on the systemic advantages of interruptions: it's bad for the patient, but perhaps good for the system. Anyway, if we look not at the systemic level and only at the individual level, interruptions should be assessed by the interrupted (12).

Currently, there are a few studies on interruptions in outpatient departments in China, mostly focused on nursing and drug delivery. Therefore, there is a need for more researches to analyze workflow interruptions in outpatient department. In other words, to optimize outpatient management, it is important to know the sources of interruptions and the relationship between interruptions and work system (9). Unlike western countries, the main body of health service delivery in China is tertiary general hospitals. They usually include outpatient department and inpatient department. This type of hospitals has to undertake one-half of the medical services of entire country. But there are some major problems in tertiary general hospitals, such as large numbers of large-scale hospitals (about 50 hospitals with more than 3,000 beds in 2019),^1^ a large number of patients and overcrowding in outpatient department. In addition, some surveys have shown that the patient experience is low in China (13). All these are challenges to hospital management. The situations that doctor deal with in the outpatient setting are complex and changeable, and the patients are also in different health conditions. Before seeing a doctor, patients will make self-judgment on their own condition or choose an appropriate department for treatment through hospital guidance. Compared with patients referred by family doctors, the process of such patients will increase the doctor's workload. Presently, the workflow of outpatient department includes problems such as the incompletely developed appointment registration system (14, 15), poor consultation process, and inadequate information applications (16), which has led to a high frequency of interruptions in outpatient department.

In this study, we observed the entire process of physicians' outpatient care using a self-made interruption observation instrument to record the frequency and sources of interruptions and determine whether the interruptions differ among specialties. We tried to understand the current situation and explore its connotation through the study of the interruption in the complex outpatient system. This is also the focus of the doctor's work system for ergonomics research in the health field.

## Methods

### Setting and Participants

This study was conducted in outpatient department of a large tertiary general hospital with average daily outpatient visits of 8,000 in Wuhan, China, in October and November 2019. This hospital is the most typical tertiary hospital in China, with a large number of outpatient visits and advanced diagnosis and treatment technology. Serving time of doctors in outpatient department is divided into morning and afternoon shifts and each shift lasts about 3 h. We chose a shift as an observation unit. The attending physician receives average 100 patients per shift, and the chief physician receives average 50 patients usually.

Surgical Medicine, Internal Medicine, Pediatrics, and Neurology are typical specialties with high physician workload and pressure. Thus, we selected the four specialties to observe physicians' workflow interruption in this study. First, all attending physicians and chief physicians with outpatient qualifications in those four specialties were included in the study. The second, we made exclusions based on physician work experience and outpatient appointments. Physicians with uncertain outpatient schedules during the study period and <1 year of work experience were excluded from the study. The last, included physicians were divided into two groups according to their professional titles: attending physicians and chief physicians. We used objective sampling to select 16 physicians from each of the two groups. In the end, a total of 32 doctors were selected, 8 in each specialty. Thirty-two physicians were invited and all agreed to participate.

The observers obtained the permission and consent of observed doctors and patients to enter the consulting room for observation and did not collect any patient-related information. This research was conducted with the permission of the Ethics Committee of Tongji Medical College of Huazhong University of Science and Technology (IORG No. IORG0003571).

### Procedure

We used the observation method, that is, to observe and record the work tasks and working hours of the observer for 1 day (about 6 h). Expert observation using standardized methods has been shown to be valid and reliable for healthcare services, with a specific application in the investigation of workflow interruption (8). Master students with 2 years of research experience in hospital management were selected as observers for this study. Before the investigation, observers receive uniform investigation training with the same material and by the same person. Observers learn to time the start and end points, learn how to identify interruptions, determine the source of interruptions and the work task.

During the investigation, an observer entered the room for continuous observation, recorded the duration of interruptions using a stopwatch, and determined the source of interruption and the type of task being interrupted. The recorded information also included the identity of patients (numbered according to the order of entering the consulting room), start and end time of interruption, source of interruption, type of work task, and remarks for special events.

### Data Collection and Measurement

In this study, a self-made observational tool was used for the investigation, and it was filled out by the observers and doctors. The tool consists of three parts: (1) the basic information of the physician, which is to be filled out by the physician after each observation; (2) the time consumption of the consultation, which is calculated and filled by the observer; (3) information on interruption, including interruption time, frequency, reason, and work tasks at the time of interruption, which the observer chooses to fill in during the observation process.

#### Observation of Work Tasks and Workflow Interruptions

**Figure 1:**
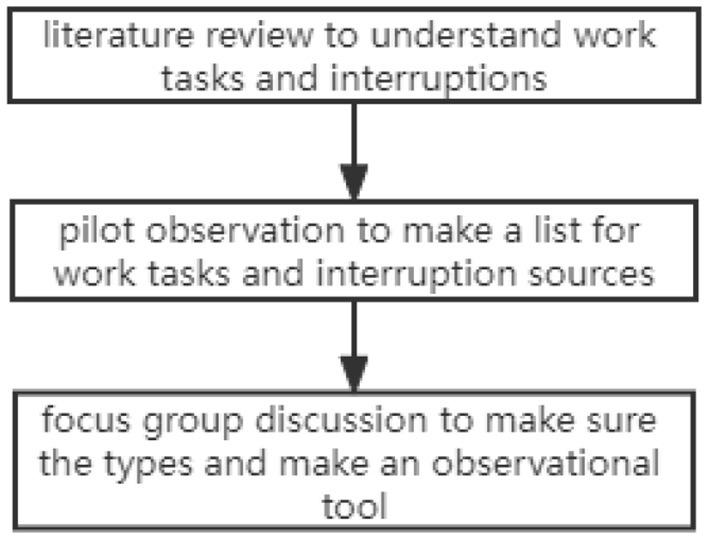
Three phases to make the observational tool.

Three phases were carried out to make the observational tool (cf. Figure 1). First, we conducted literature reviews to understand work tasks and interruptions. The second stage was to randomly select several outpatient physicians in a tertiary public hospital for pilot observation. As a result, we made a task list and an interruption source list. Finally, we invited some outpatient department managers and outpatient physicians to discuss the two lists. Based on those, an instrument was developed. Table 1 presents 8 work tasks associated with doctor-patient communication tasks and non-doctor-patient communication tasks (9, 17–19).

**Table 1:** Types of work tasks.

**Category**	**Work task**	**Definition**
Doctor-patient communication task	1. Asking health history	Inquire about the occurrence, development, current symptoms and treatment history of the disease from patients and their relatives.
	2. Explaining procedures	Explain the procedure and various precautions to patients.
	3. Diagnosing and explaining health conditions	Diagnose the patient and explain the result of examination.
	4. Giving medication precaution and health guidance	Give patients medication guidance, health guidance, follow-up matters, etc. after discharge from the hospital.
	5. Others	Other contacts between patients and doctors.
Non-doctor-patient communication task	6. Physical examination	Physical examination refers to the detection and measurement of the human body's structure and function development level.
	7. Documentation	Handle paperwork and operations including paper medical records and electronic medical records.
	8. Contacting outside	Contact the inpatient department or operating room related to patient care.

Moreover, we employed a classification system suitable for this study, which divides workflow interruptions into 11 types (cf. Table 2) (2, 17, 20–23). In this study, the closed-loop workflow of outpatient physicians is the whole work tasks and sequence from the current patient's visit to stay away from the physician's desk. Workflow interruption means that an existing work task is interrupted by an unrelated task, or an existing task is interrupted to perform tasks that are not planned. For example, when the current patient sees a doctor, an action that is interrupted by an unrelated person or business is recorded as an interruption. Different from previous research, interruption by patients or their relatives means that the current patient's process is interrupted by patients who was not visiting currently, such as people behind the queue or outside the consulting room. After we combined the recommendations of outpatient department managers and physicians and ensured that there were no missing or unsuitable classifications, an observation chart was created as an observational instrument.

**Table 2:** Sources of workflow interruption.

**Category**	**Workflow interruption source**	**Definition**
Interruption by patients or their relatives	1. Asking about medical examination results	Patients interrupt physicians to ask about the results of their physical and chemical examinations such as x-ray, blood tests, and urine tests.
	2. Asking for health guidance	Patients interrupt to ask for medical guidance, dietary restrictions, lifestyle behavior changes, and ways to improve their health when at home.
	3. Asking about the waiting time	Patients interrupt while they are queuing outside to ask when they can see a doctor.
	4. Asking about procedures	Patients interrupt to ask about procedures such as inpatient procedures and outpatient treatment procedures.
	5. Medical disputes	Patients interrupt to seek solutions due to dissatisfaction with medical results, service attitudes, outpatient procedures, etc.
	6. Others	Patients interrupt because of other issues.
Interruption by colleagues	7. Work issues	Questions about patient's condition, multi-section consultation, etc.
	8. Private issues	Issues regarding interpersonal relationships
Interruption by phone	9. Phone	An interruption because of the doctor's phone
Interruption by equipment/ system/network failure	10. Equipment/ system/network failure	Doctors are interrupted by hospital systems, network failure, medical equipment, and so on.
Interruption by others	11. Others	Interrupted by other people or other issues

#### Observational Tool: Test of Reliability

Pilot surveys were conducted to minimize the inter-observer bias. Adhering to the principle of independence and non-interference, two pre-investigations were conducted on site, with two observers simultaneously observing one doctor.

The Kappa coefficient test and the correlation coefficient test were used to test the consistency of the two sets of data. If the Kappa coefficient reaches 0.6, it is generally considered that the consistency strength is substantial (24) and the ICC value reaches 0.75, it is generally considered that the degree of consistency is good (25).

After two rounds of pilot surveys, the study finally passed the consistency tests. The record sheet and record standards would be adjusted appropriately according to the results of pilot surveys. Every item recorded was examined to verify the consistency of the tool and the familiarity of our observers with the content of the tool.

### Statistics

The data were entered into an Excel via double data entry and checked for errors and illogical values. The descriptive statistics included characteristics of outpatient department and interruptions in the hospital. Linear regression methods were used to assess outpatient characteristics as predictors of the number of interruptions. Potential risk factors for the number of interruptions included number of patients, outpatient duration, doctor title, specialties and day of the week. To assess the association between outpatient characteristics and number of interruptions, univariate analyses were performed using linear regression. The Kruskal-Wallis test was used to analyze the difference about the duration of interruptions among specialties, and the Chi-Square Test was used to examine the sources of interruptions among different specialties, to determine whether interruption source is associated with specialty. All analyses were performed using SPSS 20.0, and *p* < 0.05 was considered statistically significant for all tests.

## Results

### Characteristics of Outpatient and Workflow Interruptions

In total, we observed 32 physicians, and obtained 904 outpatient visits. Only 23 of the 32 physicians were willing to provide information such as age and doctor title. Among the 23 physicians, the male to female ratio was 7:5, and the average age was 37.9 years. A total of 320 interruptions were observed, and the total duration of interruptions was 138.82 min, which suggests that the doctors' workflow was interrupted 6.12 times per hour. Characteristics of doctors in outpatient department are shown in Table 3.

**Table 3:** Characteristics of doctors in outpatient department.

**Characteristic (per doctor)**	**Average**	**SD (Range)**
Number of patients	28	11.76 (5–59)
Number of interruptions	10	7.88 (0–35)
Duration of interruption (min)	4.3	3.54 (0–15.14)
Outpatient duration (h)	3.16	0.5 (2.58–4.22)

Characteristics of interruptions are shown in Table 4. The most common category of interruptions was interruption by patients or their relatives, followed by interruption by phone. The highest interruption rate, when stratified by work task type, occurred when doctors were asking health history: 19.95 interruptions per hour. Doctors were interrupted 14.33 times per hour when they were doing physical examination. The most of interruptions lasted less 1 min, which was account for 90%.

**Table 4:** Characteristics of workflow interruption.

**Characteristic**	***N***
**Category of interruptions** ***N*** **(%)**
Interruption by patients or their relatives	221 (69)
Interruption by colleagues	28 (8.8)
Interruption by phone	45 (14)
Interruption by equipment/system/network failure	15 (4.7)
Interruption by others	11 (3.5)
**Task being interrupted** ***N*** **(rates per hour)**
Asking health history	150 (19.95)
Explaining procedures	13 (5.23)
Diagnosing and explaining health conditions	57 (8.74)
Giving medication precaution and health guidance	23 (12.59)
Other patient-physician communication	3 (2.92)
Physical examination	10 (14.33)
Documentation	64 (6.47)
Contacting outside	0
**Length of interruption** ***N*** **(%)**
<1 min	288 (90)
1–5 min	31 (9.6)
>5 min	1 (0.4)

*N = 320, number of interruptions*.

In univariate analysis, only the number of patients was significant independent predictors of the number of interruptions. With each additional patient on outpatient, the number of interruptions increased by ~1 (coefficient = 0.76, *P* < 0.001). Outpatient duration, doctor title, specialties and day of the week were not significant independent predictors of the number of interruptions.

### Distribution of Interruptions in Different Departments

Table 5 presents workflow interruptions grouped by departments and Table 6 shows the sources of interruptions among the four specialties. We can see that all of the departments were interrupted by patients or their relatives the most. So, it was expected to explore subtypes under this category. In Internal Medicine, the most common source was interruptions by patients or their relatives because of other issues and asking about procedures, accounting for 27.5% and 17% of all interruptions, respectively. In Surgical Medicine, patients or their relatives asking about procedures (*N* = 12; 25%) was the most common interruption source, followed by interruption by patients or their relatives because of other issues (*N* = 11; 23%). In Pediatrics, the most common interruption source was patients or their relatives asking about procedures (*N* = 19; 29.7%), whereas interruptions due to medical disputes did not occur. In Neurology, the most common interruption category was interruptions by patients or their relatives because of other issues (*N* = 28; 25%), and all subtypes under the category were observed.

**Table 5:** Observed workflow interruptions grouped by the four specialties.

**Specialty**	**Number of observations**	**Number of interruptions**	**Observed time (h)**	**Interruptions per hour**	**Duration of interruption (min)**
Internal medicine	277	98	15.17	6.46	45.41
Surgical medicine	162	48	8.09	5.94	21.79
Pediatrics	210	64	14.75	4.34	23.38
Neurology	256	110	14.26	7.71	48.25

**Table 6:** Sources of interruptions among the four specialties.

**Specialty**	**Source of interruptions**				
	**Interruption by patients or their relatives: % (*N*)**	**Interruption by colleagues: % (*N*)**	**Interruption by phone: % (*N*)**	**Interruption by equipment/system/network failure: % (*N*)**	**Interruption by others: % (*N*)**
Internal medicine	65.3 (64)	7.1 (7)	12.3 (12)	8.2 (8)	7.1 (7)
Surgical medicine	75 (36)	4.2 (2)	16.6 (8)	2.1 (1)	2.1 (1)
Pediatrics	65.6 (42)	14.1 (9)	10.9 (7)	7.8 (5)	1.6 (1)
Neurology	71.8 (79)	9.1 (10)	16.4 (18)	0.9 (1)	1.8 (2)

*N, number of interruptions*.

A Kruskal-Wallis test analysis demonstrated statistically significant difference in sources of interruptions based on four specialties (*X*^2^ = 16.988, *p* = 0.049). Hypothesis testing was applied to examine the difference in the duration of interruptions among the four specialties. It was found that the duration of interruptions was not significantly different among the four specialties (*X*^2^ = 2.732, df = 3, Kruskal-Wallis = 0.435), but the difference about the duration of interruptions by phone, equipment/system/network failure, and other interruptions were significantly different among specialties (*X*^2^ = 8.324, df = 3, Kruskal-Wallis = 0.04). A pairwise comparison of the significance level adjusted by Bonferroni found that the difference between Pediatrics and Surgical Medicine (*X*^2^ = −2.795, Kruskal-Wallis = 0.031) was statistically significant, but there was no statistically significant difference between Pediatrics and Internal Medicine, Pediatrics and Neurology, Internal Medicine and Surgical Medicine, Internal Medicine and Neurology, Surgical Medicine and Neurology.

## Discussion

The outpatient environment in China is complex with short-term communication and frequent task changes, which impose significant demands upon outpatient physicians. In order to understand the working environment and high workload of outpatient physicians, it is necessary to comprehensively consider the relationship between patients, doctors, and hospital management. Workflow interruption is one of the influencing factors of workload (21). In this vein, we set out to study the outpatient workflow interruption and the differences in the distribution of interruptions among different specialties in a typical tertiary hospital in China. In terms of our results, this study contributes to the current research on doctors' workflow interruption from the following aspects.

First, this study recorded and quantified the physician's workflow interruptions from four different clinical specialties in outpatient department of tertiary general hospitals in China through standardized observations, which enriched the evidence of the physician's workflow interruption with outpatient care and various clinical departments. A considerable amount of literature has been published to show the interruptions about inpatient care and few is about outpatient care. In our research, the physicians' outpatient workflows were interrupted overall approximately 6.12 times per hour, which was lower than that in emergency department (17, 26). Among the four clinical specialties we observed, Neurology was the specialty with the most frequent interruptions of 7.71 times per hour. Regarding interruption sources, our study showed that the majority of sources was attributed to patients and their relatives, which is quite different from previous studies that were mostly attributed to intra-departmental communication (23, 27, 28) and telephone/beeper calls (29). The reason may lie in the queuing management of the visiting patients during physicians' consultation in China. There are some patients queuing in the consulting room in outpatient department and some patients who return to the room after obtaining the test/examination result or the prescribed medicine can enter the consulting room directly without the queuing number. These may be potential risks for the patient to interrupt the doctor (30). Regarding the physicians' work tasks being interrupted, the rate per hour of being interrupted during asking patients' health history is higher than that during other work tasks, which may be the result of frequent change of consulting patient and the physicians' consultation usually begins with the task of asking patients' health history. When the doctor resumes talking to the patient, they may forget to ask something or repeat the questions (31). These interruptions disrupt the communication so that the doctor's attention cannot be focused on the current patient's consultation. This interferes with the thought process and increases cognitive demands, thereby increasing the risk of errors (23). The difference in the interruption sources among different clinical specialties mainly stems from the characteristics of specialties (28). Different clinical specialties have different outpatient procedures, and the conditions of patients in these specialties are different.

Second, our study discussed and analyzed how physicians' workflow interruptions operated in the complex work system. The outpatient care in Chinese tertiary general hospital is characterized with large patient flow and frequent task switching of physicians. Physicians in outpatient care work for nearly 3 h per half day and have limited range of motion. Therefore, we observed that part of interruptions occurred actively by physicians themselves. Workflow interruption can be an adaptive response of doctors in an outpatient setting, which is similar to other interruption studies (32–34). In some studies, the interruption is to obtain more information to better deal with the patients' condition, or is the response strategy of the doctor to deal with a certain prompt (26). For example, queuing in the consulting room is chaotic, and doctors suspend work tasks to maintain visit order. Or, in order to improve the work efficiency, doctors will actively choose to be interrupted when dealing with documentation, and perform multitasking simultaneously (35). Task of differing modalities, in this case verbal and non-verbal, can more easily be performed in parallel than tasks of the same modality (26). Actually, doctors will reduce the impact of interruption by multitasking, ignoring or deferral. This kind of active interruption can effectively increase the working efficiency of physicians while they can also increase the physicians' workload (36). Previous study have showed that task-switching introduces risk of no-resumption or resuming at the wrong place in a task sequence (26). Moreover, a non-interrupted process is also a requirement of the hospital to realize the patient-centered concept and to improve the patient experience. Therefore, the most important management measure is to optimize the physician's outpatient working environment. For example, it is helpful to limit the number of patients entering the consulting room (23). In order to reduce the physicians' interruptions by patients due to non-medical issues, assistants can be arranged outside the consulting room or in a waiting area of patients to solve such problems (37).

In this study, the rate per hour of interruptions during doctor-patient communication task was much higher than that during non-doctor-patient communication task. This phenomenon is different from the previous study (26). In this regard, it should be attributed to the patient's consciousness and the fact that doctors tend to have more time during doctor-patient communication tasks than that during non-doctor-patient communication tasks (38). For example, patients believe that doctors are more likely to accept interruptions during doctor-patient communication tasks and shift doctors' attention to themselves. Therefore, the environment, not only the physical environment but also hospital outpatient service system and hospital management measures, plays an important role in understanding the occurrence of interruptions (9, 39). When discussing the high workload of physicians, interruption, as an intermediate product, is the result of the dynamic action of people and the system. When it comes to one outpatient shift, doctors tend to complete the daily consultations by taking care of patients quickly and attentively. Under this circumstance, interruption may be repeated many times in a short period of time, also known as “nested interruption”, which will increase the mental workload of providers and influence their subjective cognition (38).

Third, the results of our study are generalized and practical. The observed hospital is a typical tertiary general hospital in China. According to China's 2020 Health Statistics Yearbook, there were about 2,749 tertiary hospitals in China, accounting for 8% of all hospitals, but they had to undertake one-half of the health services of entire country. This type of hospitals has advanced technology and high-quality services, and usually is the first choice for patients to seek medical treatment in China. Although we only conducted observations in this hospital, the results are widely applicable to most tertiary hospitals in China and also to the same scale hospitals with large number of patients in the world. The high frequency of interruptions and complex sources of interruptions revealed by our study are common problems in many hospitals. Therefore, the interruption management strategies and hospital management measures proposed in our study could be references for most of the hospitals and have great practicability and generalization.

In this study, the specific connotation of interruption is based on the judgment of patient-centered. It should be a complete process from entering the consultation room to leaving the consultation room to patients, and any person or event that has nothing to do with his/her disease is interruption. But if we only look at the interruption from one angle, our discussion tends to be one-sided (40). Interruption by patients or their relatives is bad for the current visiting patients, but it can help patients who interrupt the workflow achieve more medical information to improve medical effects. To reduce these interruptions by patients or their relatives, we recommend to set up medical assistants in outpatient department to answer the questions of returning patients. In this way, they can achieve information not interrupting the doctors who is visiting the other patient. Interruption by phone and interruption by equipment/system/network failure are also common in our study. This interruption is more random than interruption by colleagues and patients, because colleagues and patients are able to pick a time when it is appropriate to interrupt (27). Previous research has shown that “random” interruptions are more strongly related to work impairment than “reflexive” interruptions (9). Moreover, phone-related prompt and interruptions are always easier to cause task switching, which means they have a higher priority (27). In order to reduce “random” interruptions, electronic assistance and regular maintenance may be a promising approach (41).

### Limitations

In this study, a typical hospital was chosen to represent the current status and characteristics of outpatient services in China. However, tertiary public hospitals are the main body of health service delivery in China. This type of hospital provides most of health services, and has strong medical strength and patient trust. Therefore, the study reflected the actual situation of interruptions in outpatient department in China to a certain extent, and had broad applicability and generalizability. Second, based on the requirements of the hospital used for the observations, all physicians observed in this study were limited by hospital outpatient arrangement; thus, the study was incomplete random sampling. Therefore, we asked the manager to try their best to select randomly without taking particular factors about the doctors into consideration. The research method was the observation method, and limitations to the study include those inherent to observational studies. Although the observers had been trained beforehand on the content of the observations and the precautions to be taken, there remained a great potential for bias between theory and the understanding of observers, which was unavoidable. For the observed physicians and patients, although measures were taken to reduce the risk of the Hawthorne effect [the observer stood at least 1 meter away from the physician in the clinic; during the study, the investigator did not actively talk to the subject (17)], and tried not to explain our research and observation entries before the study began, it still cannot be ruled out that the doctors may have inferred our observations, thus modifying their responses accordingly.

### Implications for Research and Practice

Our investigation is helpful to the growing research on interruptions based on the outpatient work systems, and to explore the internal reasons for the difference about interruptions among different clinical specialties. In an outpatient setting, interruption is inevitable and necessary to a certain extent. In view of the large number of outpatient visits and the poor hospital management, future research should explore the significance and impact of outpatient workflow interruptions, and how to reduce unnecessary interruptions while ensuring the efficiency of the outpatient. Since its potential impact depends on the patient and the environment, interruption resolution strategies to improve the workload of doctors should be considered in the wider hospital system. This refers particularly to interruptions during important diagnostic tasks that are passive or ineffective. This study also provides information for future research through discussing the possibility of interruptions among different departments and processing strategies for interruptions in a complex work system, so as to improve the doctor's workload, reduce decision-making errors and improve patient safety.

Regarding the impact of the interruption on outpatient practice, our results call for further efforts in the design of hospital management and outpatient work systems to improve the workload of doctors. Since interruption is inevitable to some extent, and even needs to exist. Therefore, it is necessary to identify the meaning and impact of the interruption. Establish a full-process team through hospital outpatient management to reduce interruptions caused by queuing, waiting and other chores. Outpatient doctors work in complex and dynamic situations. Thus, it is expected to improve the doctor's non-technical skills and the flexible design of outpatient management, which can be adjusted according to the patient's situation at any time. In addition, the participation and promotion of the whole society is beneficial to improving the health literacy of patients.

## Conclusion

The observation of the entire outpatient process allowed a more detailed and clear understanding of workflow interruptions, which will lay the foundation for further research on the effects of interruptions on physicians and patients. Our findings further prove that there are certain differences about the interruption among clinical specialties, not limited to the departments with obvious differences like Emergency Department and Primary Care Department. Our research results increase the knowledge on interruptions of outpatient department, and provide a basis for future research about the fields of tasks, clinical specialties and hospital managements.

## Data Availability Statement

The raw data supporting the conclusions of this article will be made available by the authors, without undue reservation.

## Ethics Statement

The studies involving human participants were reviewed and approved by the Ethics Committee of Tongji Medical College of Huazhong University of Science and Technology (IORG No. IORG0003571). The patients/participants provided their written informed consent to participate in this study.

## Author Contributions

XZ, YH, LW, and DL conceived and designed the study. XZ, DL, LW, SX, SC, and XW collected data. XZ analyzed data and drafted the manuscript. All authors provided constructive suggestions to improve the paper and approved the final version of the paper.

## Funding

This study was supported by the National Natural Science Foundation of China (Grant Number 71774062) and the Fundamental Research Funds for the Central Universities (Grant Number 2021WKYXZX008).

## Conflict of Interest

The authors declare that the research was conducted in the absence of any commercial or financial relationships that could be construed as a potential conflict of interest.

## Publisher's Note

All claims expressed in this article are solely those of the authors and do not necessarily represent those of their affiliated organizations, or those of the publisher, the editors and the reviewers. Any product that may be evaluated in this article, or claim that may be made by its manufacturer, is not guaranteed or endorsed by the publisher.

## Footnotes

^1^China's 2020 Health Statistics Yearbook.
